# A Case of Sinus of Valsalva Aneurysm Rupture Causing Cardiogenic Shock

**DOI:** 10.7759/cureus.44210

**Published:** 2023-08-27

**Authors:** Sriharsha Dadana, Anusha Kondapalli, Vipul Madhwani

**Affiliations:** 1 Internal Medicine, Cheyenne Regional Medical Center, Cheyenne, USA; 2 Cardiology, Cheyenne Regional Medical Center, Cheyenne, USA

**Keywords:** right sinus of valsalva, cardiac aneurysm, cariogenic shock, cardio vascular surgery, sinus of valsalva aneurysm

## Abstract

A sinus of Valsalva aneurysm (SVA) is an abnormal dilation between the aortic valve annulus and sinotubular junction resulting from weakness in the elastic lamina. In the vast majority of cases, SVAs are asymptomatic and are incidentally detected on echocardiogram imaging. In some cases, they can rupture and lead to an intracardiac shunt. Sudden rupture of a high-flow aneurysm can lead to significant hemodynamic compromise and a high fatality rate if not diagnosed early and intervened upon. We present the case of a 68-year-old male who presented with symptoms of heart failure and later rapidly deteriorated due to a sudden spontaneous rupture of the SVA leading to cardiogenic shock. In our case, timely identification and intervention led to a good outcome for our patient. We also present echocardiogram images and videos to educate the readers further about diagnosing SVA. With this case report, we would like to help clinicians and researchers expand their understanding of the condition and treatment outcomes.

## Introduction

A sinus of Valsalva aneurysm (SVA) is a rare congenital or acquired anomaly caused by any conditions that lead to weakness of the elastic lamina at the junction of the aortic media and the annulus fibrosis, such as connective tissue diseases; infections such as syphilis, tuberculosis, or endocarditis; or a congenital condition seen in about 0.09% of the general population [1,2]. The SVA was first described by John Thurman in 1840 [3]. Typically, men are more affected, and there is a higher reported incidence in Asian groups. Most SVAs are diagnosed based on echocardiography, with or without angiography [4]. The SVAs usually affect the right coronary sinus, followed by the noncoronary sinus, and finally the left coronary sinus [5,6]. Here, we present a rare case of a 68-year-old male with a right SVA rupture leading to cardiogenic shock and needing urgent repair. While SVA rupture carries a high mortality and morbidity rate, prompt diagnosis and treatment have also been shown to have excellent outcomes with a good long-term prognosis.

## Case presentation

The patient is a 68-year-old male with a history of coronary artery disease with prior percutaneous coronary intervention (PCI) 23 years ago and hypertension. He presented to the emergency department with complaints of exertional dyspnea, orthopnea, leg swelling, and jaw pain. The patient had not been seeing any providers for many years prior to the current presentation, and as a result, prior echocardiogram or cardiac catheterization information was unavailable. On exam, there was elevated jugular vein distention (JVD) at 20 cm, tachycardia with a heart rate of 115 beats/min, normal S1 and S2 with a 6/6 holosystolic murmur at the apex and S3 gallop, bilateral rales on lung exam, and pitting edema. Labs showed elevated troponin at 0.05 ng/ml (reference range: < 0.03 ng/ml) and elevated beta-natriuretic peptide at 4360 pg/ml (reference range: <300 pg/ml). Creatinine was 1.1 mg/dl (reference range: <1.2 mg/dl), and his liver function tests were aspartate aminotransferase (AST)/alanine transaminase (ALT) at 37/45 U/l (reference range: 10-45 U/l). The electrocardiogram showed sinus tachycardia with non-specific ST-T wave changes. A chest X-ray showed bilateral pleural effusions with pulmonary vascular congestion. The patient was started on aspirin, atorvastatin, metoprolol XL, and heparin drip for presumed acute coronary syndrome, and intravenous furosemide for heart failure.

A transthoracic echocardiogram (TTE) showed a normal ejection fraction of 65% with inferoseptal hypokinesis and an abnormal tricuspid valve septal leaflet appearing to have a cyst or other fluid-filled structure with moderate tricuspid regurgitation (TR). A high-velocity jet seemed to interfere with the tricuspid valve opening, and it felt like there may be a small membranous ventricular septal defect (VSD). Unfortunately, we didn't have any prior images or reports to compare.

Coronary angiography showed ostial left main (LM) at 10% to 20%, ostial-prox LM left anterior descending artery (LAD) at 20%, mid (m)LAD artery at 30% to 40%, left circumflex artery (LCx) and proximal left circumflex (pLCx) at 70%, in-stent restenosis (ISR) and first obtuse marginal artery (OM1) at 99% subtotal occlusion in the mid-third right coronary artery (RCA), and mRCA chronic total occlusion (CTO). There was also evidence of mild pulmonary hypertension with sats of 85% in the pulmonary artery (PA), suggesting a left-to-right (L-to-R) shunt. The shunt was felt to be most likely below the level or at the distal end of the right atrium (RA), as the mRA pressure was normal.

**Figure 1:**
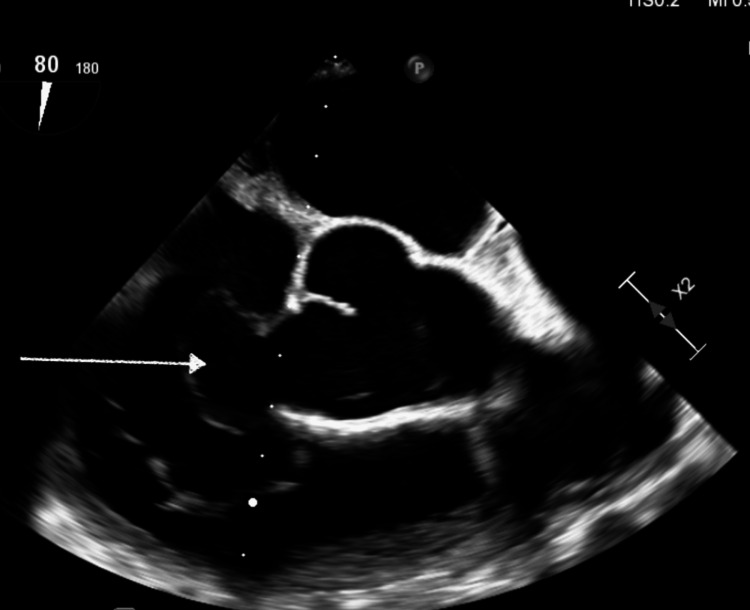
TEE image showing right SVA (arrow) TEE: Transesophageal echocardiogram, SVA: Sinus of Valsalva aneurysm

**Figure 2:**
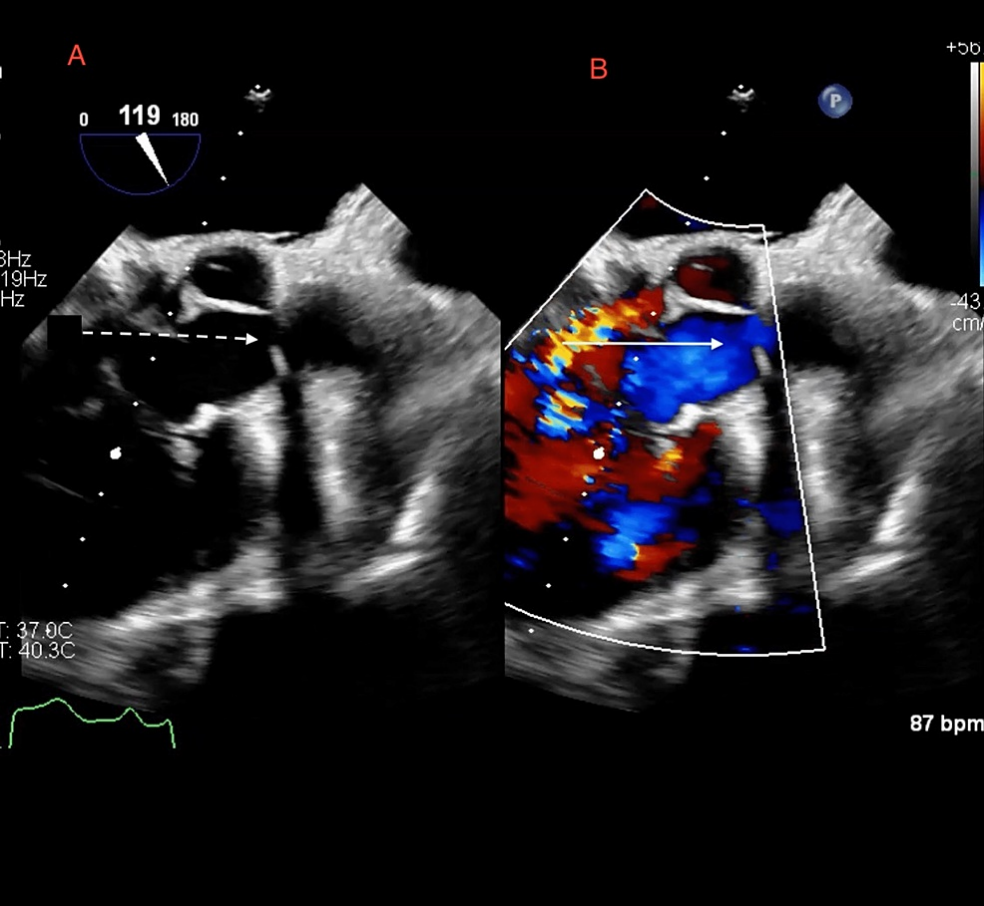
TEE images A: Visualization of the right SVA (dotted arrow),  B: Color Doppler showing intra-cardiac shunting at the site of SVA rupture (straight arrow) TEE: Transesophageal echocardiogram, SVA: Sinus of Valsalva aneurysm

A transesophageal echocardiogram (TEE) showed a right SVA with communication between the right aortic sinus and RA and grade 1 diastolic dysfunction (Figure 1, Video 1). A TEE with color Doppler showed the presence of SVA rupture with an L-to-R intracardiac shunt (Figure 2, Video 2).

**Video 1:** TEE video showing right SVA with communication of right aortic sinus and right atrium TEE: Transesophageal echocardiogram, SVA: Sinus of Valsalva aneurysm

**Video 2:** TEE video with color Doppler showing the presence of SVA rupture with left-to-right intra-cardiac shunt TEE: Transesophageal echocardiogram, SVA: Sinus of Valsalva aneurysm

The patient suddenly had a presyncope episode with abdominal pain, vomiting, diaphoresis, hypotension (BP 89/42 mmHg), confusion, and agitation. Labs revealed new-onset acute kidney injury with blood urea nitrogen (BUN) of 42, creatinine at 2.8 mg/dl, and acute liver injury with ALT at 1325 U/L and AST at 2675 U/L. Blood gas revealed a pH of 7.33, a partial pressure of carbon dioxide (pCO2) of 25, a partial pressure of oxygen (pO2) of 96, and bicarb of 13 mmol/L (reference range: 22-28 mmol/L). 

Cardiothoracic surgery was consulted. The patient was started on a dobutamine drip for cardiogenic shock, but he continued to decompensate. Hence, he was taken urgently for repair of the right SVA with an aorto-atrial fistula, coronary artery bypass graft (CABG) saphenous vein graft (SVG) to OM1, and left atrial appendage ligation. Post-intervention, an intraoperative echocardiogram revealed good function of the aortic valve with no significant regurgitation. Preoperative trace regurgitation was slightly improved. No obvious wall motion abnormalities were noted.

Post-repair of the right coronary SVA, the patient developed cardiac conduction disease and a third-degree heart block. Repair of this aneurysm resulted in transection of the cardiac conduction system and third-degree heart block without ventricular escape, which was initially managed by an epicardial pacing lead. He eventually underwent permanent dual-chamber pacemaker placement. The patient was slowly weaned off of vasopressor support. His liver function and renal function continued to improve, and he was discharged on postoperative day 7.

The TTE on the follow-up visit showed moderately reduced left ventricular systolic function with an ejection fraction of 44%, no aortic valve stenosis or regurgitation, mild pulmonary hypertension, moderate tricuspid valve regurgitation, moderate pulmonic regurgitation, and normal RA pressure. The patient has regular clinic follow-ups, is compliant with goal-directed medical therapy, and has finished cardiac rehab. He is being followed up in the cardiology clinic every three months, and an echocardiogram will be obtained annually.

## Discussion

Generally, small, unruptured SVAs are asymptomatic and are only incidentally found. An SVA rupture into the cardiac chambers results in continuous shunting from the aorta to the receiving chamber. This results in patients presenting with acute heart failure symptoms like those seen in our patient [7]. In the case of a right or noncoronary sinus aneurysm rupture, a fistula forms between the aorta and the RA or RV, causing L-to-R shunting. On the other hand, a rupture in the left sinus aneurysm leads to fistulous communication between the aorta and the left atrium or left ventricle [8]. Furthermore, SVAs can also be complicated by aortic regurgitation in about 20% to 30% of cases [9]. Tricuspid stenosis or regurgitation, as seen in our patient, has also been reported [10]. Based on our patient's presentation, his sudden decompensation to cardiogenic shock was likely due to the progression of his rupture leading to a worsening shunt. We also believe that spontaneous rupture was likely the etiology given that the patient was hemodynamically stable with well-controlled blood pressures prior to acute decompensation.

Both TTE and TEE are fast, non-invasive ways to assess the characteristics of aneurysmal dilatation, cardiac chamber involvement, and valvular assessment, as well as any associated cardiac anomalies [11]. In the context of SVA rupture, spectral color Doppler imaging will reveal a persistent turbulent flow between the aneurysm and the receiving chamber and is crucial to aiding diagnosis. As seen in our case, initial TTE led to an erroneous diagnosis of the patient likely having VSD, and ultimately TEE with color Doppler aided in getting an accurate diagnosis. Though both cardiac CT and cardiac magnetic resonance (CMR) offer precise visualization of cardiac anatomy, it is CMR that minimizes radiation exposure while providing superior contrast between tissues and blood vessels [12]. Cardiac catheterization is commonly conducted to assess coronary anatomy before surgery, and as seen in our patient, elevated PAO2 saturation also indicates the presence of an L-to-R shunt.

The mortality rate for ruptured SVA is alarmingly high. Surgical repair is associated with a low mortality rate ranging from 1% to 7% and demonstrates excellent long-term survival rates, even when dealing with cases of rupture. In our patient, given the need for CABG, a surgical approach was considered to be ideal to obtain good outcomes. Furthermore, the likelihood of SVA recurrence is relatively uncommon with surgical intervention [13,14]. As seen in our case, with surgical intervention, sometimes cardiac conduction anomalies can be noted, and heightened vigilance and monitoring are needed. Alternative treatment includes utilizing percutaneous closure repair using occluders given their minimally invasive approach, although they are mostly considered in patients with small-sized ruptures and no associated cardiac abnormalities. Some studies have shown similar outcomes in both surgical and percutaneous interventions [15]. 

## Conclusions

An SVA is a rare cause of cardiogenic shock and carries high morbidity and mortality if not promptly diagnosed and treated. As seen in our case, it is well established that timely intervention leads to excellent outcomes on follow-up. Our case also highlights the utility of echocardiography, especially TEE, in diagnosing SVA. While surgical intervention is still the treatment of choice, further studies to assess the effectiveness of the percutaneous closure approach are still needed.

## References

[1] Hope J (1840). A treatise on the disease of the heart and great vessels, and on the affections which may be mistaken for them. Med Chir Rev.

[2] Shumacker HB Jr (1972). Aneurysms of the aortic sinuses of Valsalva due to bacterial endocarditis, with special reference to their operative management. J Thorac Cardiovasc Surg.

[3] Abuchaibe EC, Dobrolet N, Peicher K, Ventura R, Welch E (2012). Sinus of Valsalva aneurysm rupture: an unusual presentation of chromosome 22q11.2 deletion: a case report. Case Rep Pediatr.

[4] Yang Y, Zhang L, Wang X (2017). Echocardiographic diagnosis of rare pathological patterns of sinus of Valsalva aneurysm. PLoS One.

[5] Feldman DN, Roman MJ (2006). Aneurysms of the sinuses of Valsalva. Cardiology.

[6] Chu SH, Hung CR, How SS (1990). Ruptured aneurysms of the sinus of Valsalva in Oriental patients. J Thorac Cardiovasc Surg.

[7] Reichert CL (2008). Ruptured sinus Valsalva aneurysm, a rare cause of heart failure. Neth Heart J.

[8] Weinreich M, Yu PJ, Trost B (2015). Sinus of valsalva aneurysms: review of the literature and an update on management. Clin Cardiol.

[9] Lutaaya M, Rajagopal R, More RS (2013). Giant unruptured Sinus of Valsalva aneurysm: an unusual cause of aortic regurgitation. Heart.

[10] Gibbs KL, Reardon MJ, Strickman NE (1986). Hemodynamic compromise (tricuspid stenosis and insufficiency) caused by an unruptured aneurysm of the sinus of Valsalva. J Am Coll Cardiol.

[11] Johari MI, Deraman HMA, Mohamed MS, Daud BMA (2021). An unusual cause of recurrent syncope: sinus of Valsalva aneurysm. Cureus.

[12] Hanna MF, Malguria N, Saboo SS, Jordan KG, Landay M, Ghoshhajra BB, Abbara S (2017). Cross-sectional imaging of sinus of Valsalva aneurysms: lessons learned. Diagn Interv Radiol.

[13] Takach TJ, Reul GJ, Duncan JM (1999). Sinus of Valsalva aneurysm or fistula: management and outcome. Ann Thorac Surg.

[14] Vural K (2001). Approach to sinus of Valsalva aneurysms: a review of 53 cases. Eur J Cardiothorac Surg.

[15] Kuriakose EM, Bhatla P, McElhinney DB (2015). Comparison of reported outcomes with percutaneous versus surgical closure of ruptured sinus of Valsalva aneurysm. Am J Cardiol.

